# Blood Vessel Adaptation with Fluctuations in Capillary Flow Distribution

**DOI:** 10.1371/journal.pone.0045444

**Published:** 2012-09-27

**Authors:** Dan Hu, David Cai, Aaditya V. Rangan

**Affiliations:** 1 Department of Mathematics, MOE-LSC, and Institute of Natural Sciences, Shanghai Jiao Tong University, Shanghai, China; 2 Department of Mathematics, MOE-LSC, and Institute of Natural Sciences, Shanghai Jiao Tong University, Shanghai, China; 3 Courant Institute of Mathematical Sciences and Center for Neural Science, New York University, New York, New York, United States of America; 4 Courant Institute of Mathematical Sciences, New York University, New York, New York, United States of America; University of Zurich, Switzerland

## Abstract

Throughout the life of animals and human beings, blood vessel systems are continuously adapting their structures – the diameter of vessel lumina, the thickness of vessel walls, and the number of micro-vessels – to meet the changing metabolic demand of the tissue. The competition between an ever decreasing tendency of luminal diameters and an increasing stimulus from the wall shear stress plays a key role in the adaptation of luminal diameters. However, it has been shown in previous studies that the adaptation dynamics based only on these two effects is unstable. In this work, we propose a minimal adaptation model of vessel luminal diameters, in which we take into account the effects of metabolic flow regulation in addition to wall shear stresses and the decreasing tendency of luminal diameters. In particular, we study the role, in the adaptation process, of fluctuations in capillary flow distribution which is an important means of metabolic flow regulation. The fluctuation in the flow of a capillary group is idealized as a switch between two states, i.e., an open-state and a close-state. Using this model, we show that the adaptation of blood vessel system driven by wall shear stress can be efficiently stabilized when the open time ratio responds sensitively to capillary flows. As micro-vessel rarefaction is observed in our simulations with a uniformly decreased open time ratio of capillary flows, our results point to a possible origin of micro-vessel rarefaction, which is believed to induce hypertension.

## Introduction

The blood vessel system of animals and human beings is efficient in regulating blood flow according to metabolic demand of the tissue. In response to a short-term change of tissue states, metabolic flow regulation processes of the blood vessel system are able to supply sufficient blood to tissues within a few seconds. The metabolic flow regulation processes include the regulation of blood pressure, the dilation or contraction of blood vessels, and fluctuations in the capillary flow distribution [1]–[6]. Meanwhile, in response to a long-term change of tissue metabolic demand, chronic changes of blood vessel structures are also able to meet the changes in blood delivery [7]. The chronic adaptation processes include structural changes of luminal diameters [8]–[10], remodeling of vessel walls (the change of vessel wall thickness without any change in wall mass [11]), and generation and degeneration of micro-vessels [12]. The chronic adaptation is crucial for physical growth of individuals and their adaptation to their environment. However, some diseases are also closely related to the adaptation, such as hypertension and ischemic heart diseases [8], [11]–[13]. Therefore, it is important to study the chronic adaptation of blood vessel systems in order to achieve a clear understanding of the pathogenesis of these diseases.

The blood vessel system can achieve an efficient blood delivery within seconds by different means of metabolic flow regulation. First, some proteins in the aorta can sense the concentration of carbon dioxide (CO_2_), sodium ions (Na^+^), etc. In response to the change of these concentrations, the nervous system can modulate the heart pressure to supply more (or less) blood to the body. Second, the vessels can sense the change of the wall shear stress by endothelial cells, which are the inner layer of the blood vessel walls. The response to these changes gives rise to regulation of the luminal size within a few seconds [14]–[17]. This regulation is achieved by contraction or relaxation of smooth muscle cells, which are the middle layer of vessel walls. The regulation can strongly control the peripheral resistance of the blood vessel system. Finally, the circulation system can regulate the distribution of capillary flows within a few seconds in response to the state of the tissue, such as concentrations of CO_2_, O_2_, Na^+^, and K^+^ [1]–[6], [18]. As has been suggested in the work of [5], this regulation is achieved by terminal arterioles and possibly the postcapillary vasculature. The contraction of a terminal arteriole leads to a decrease of the flow in its downstream capillary group or even closes the downstream capillary flow. As a result of changes in the capillary flow distribution, blood flow in large arteries is also changed. For example, when the concentration of O_2_ becomes low and that of CO_2_ becomes high in the tissue, the heart pressure increases, the blood vessels dilate, and the probability for capillary flows to switch on increases. As a result, more blood is delivered to the tissue to supply O_2_ and remove CO_2_. It has also been shown in experiments that the vessels can also regulate their diameters by the circumferential wall tensile stress, which is produced by the pressure jump across the vessel wall [9], [15]. For example, when the blood pressure is higher than normal in the arteries, the vessel wall becomes thicker, while the luminal diameter decreases and the peripheral resistance increases. As a result, the blood pressure in capillaries is maintained at a level so as to prevent the capillaries from damage.

Chronic changes, such as the growth of tissues, the alteration of metabolic consumption (e.g., through long-term physical exercises), and the change of living conditions (e.g., the altitude), have been observed to induce adaptive structural changes in vessel diameter, wall thickness, and micro-vessel density [19]–[25]. About one century ago, Thoma [26] noticed a cubic relation between the diameters of the parent vessel and its daughter vessels. A few decades later, Murray [27] used a minimal power principle to explain this relationship and found that the optimal wall shear stress is uniform for all the vessels. This conclusion is consistent with later experimental observations [7], [28] – the wall shear stress varies within about one order of magnitude along the circulation system. As suggested in the works of [8], [9], a possible origin of the differences in wall shear stress comes from the adaptation to blood pressure. Murray’s law also suggests that the relatively uniform wall shear stress along the circulation system is a consequence of the chronic adaptation. As in the metabolic flow regulation, the circumferential wall tensile stress also plays an important role in the chronic adaptation. For example, arteries in hypertension patients are found to have thicker vessel walls in small arterioles [9], [11], [29]. It is believed that this phenomenon is the result of chronic adaptation to the high blood pressure.

Based on the fact that blood vessels can sense the wall shear stress by their endothelial cells, regulate their luminal diameters by the wall shear stress, and adapt their diameters to maintain a relatively uniform wall shear stress, it has been naturally assumed in previous studies [9], [30] that there is a chronic adaptation of the luminal diameter to the wall shear stress. However, it is also found that the adaptation to local shear stress alone is not sufficient to maintain a stable equilibrium of the blood vessel trees [30]–[32]. Although the adaptation to the circumferential wall shear stress is also an important process, it is not sufficient to account for the stability [9]. To address this stability issue, in recent works of [8]–[10], [33], [34], it has been realized that the vessel system must meet the metabolic demand. In the works of [8]–[10], a reference blood flow rate for each vessel is used to characterize the metabolic demand. Additional metabolic stimulus are introduced to the adaptation process based on the metabolic demand in the works of [8]–[10], [33], [34]. By these assumptions, the resulting system becomes locally stable. Nevertheless, the questions of how the large vessels sense their reference flow rates and how the additional metabolic stimulus is generated remain to be answered and there needs further experimental evidence for such processes. Therefore, what is the mechanism that stabilizes the vessel adaptation remains an interesting question and deserves a further investigation.

To address this question, in this work, we propose a minimal model for the adaptation of blood vessel systems. In this model, we will neglect some of the detailed physiological processes that are not important to the stability of the adaptation. As in the previous works of [8]–[10], [33], [34], we also assume that the adaptation must meet the metabolic demand. However, instead of introducing an additional metabolic stimulus, we take into account the effects of metabolic flow regulation, most importantly, the change in the distribution of capillary flows. As suggested in the work of [5], capillaries are fed in groups (with the size of 10–20 capillaries) by a single terminal arteriole. In our model, capillary groups are idealized to have two possible states – open and close states, which characterize fluctuations in the capillary flow distribution. An Open Time Ratio (OTR) which is the probability for a capillary group to be open is introduced and assumed to describe the response to the tissue state. As will be shown below, the model system of chronic adaptation becomes locally stable after incorporating the effects of this open-close switch. As will be mentioned below, the graded control of capillary flows is expected to have similar effects to that of the idealized open-close switch in generating a locally stable chronic adaptation. It is important to pursue the investigation of detailed effects of graded responses in future studies. However, we will mainly focus on the open-close switch of capillary groups. In our model, we do not explicitly invoke metabolic demand as stimulus. Instead, we assume that the metabolic flow regulation gives rise to changes in both the blood flow rate and wall shear stress. This process, in turn, induces an effective additional stimulus. In the following, it will be further shown that, the OTRs and the sensitivity of capillary flows introduced in the model may be important for understanding the pathogenesis of vascular diseases, such as hypertension. As is well known, micro-vessel rarefaction may induce hypertension [9], [11], [29]. However, the detailed origin of the micro-vessel rarefaction remains to be elucidated. In our minimal model, a global decrease of the OTRs can induce a micro-vessel rarefaction. This observation may provide some insight to the relationship between the behavior of OTR and hypertension.

### Previous Studies and the Stability Issue

We first briefly describe some important physiological facts about the blood vessel system. We then review a model [8], [30] of chronic adaptation of vessels, which takes into account these physiological facts, and recall the stability property of the model for simple vessel systems. This model will be a starting point for our study.

#### Resistance and wall shear stress

For small vessel trees (e.g., for those whose luminal diameters are smaller than 0.6 mm), the Reynolds number is very low. As a result, the blood flow within a small vessel is well approximated by the Poiseuille flow [8]. In addition, the vessel wall can be viewed as a fixed cylinder because fluctuations of the blood pressure are small. Under these approximations, for a single vessel, we can obtain the volumetric flow rate *Q* and the wall shear stress 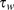


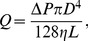
(1)

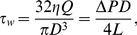
(2)where 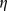 is the blood viscosity, 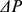 is the pressure drop, 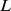 is the vessel length, and 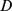 is the luminal diameter. By an analogy to an electrical circuit, the resistance 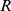 of the vessel can be defined as



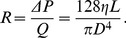
(3)Note that the resistance depends only on the length and the diameter of the vessel and is very sensitive to the change of diameter because of the inverse quartic power in Eq. (3). As a result, the vessel system can regulate the blood flow efficiently by controlling luminal diameters. Clearly, for large vessels, the fluid dynamics of blood flow can be much more complex.

We now consider complicated vessel systems with many vessels, such as an arterial tree or a network with arteries, capillaries and veins. For a given pressure drop 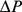, luminal diameters 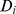, and vessel lengths 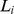 of each vessel 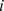, all the quantities (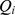, 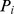, 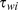) can be easily obtained by an analogy of the blood vessel system to a corresponding electrical circuit [8], in which, the blood pressure corresponds to the voltage, the flow rate to the electrical current, and the vessel resistance to the electrical resistance. Eq. (3) can be used to calculate the resistance of all the vessels. As in an electrical circuit, Kirchoff’s law can be invoked to determine the blood flows and pressures in all vessels. Finally, Eq. (2) can be used to calculate the wall shear stress.

#### Vessel adaptation to wall shear stress

As is mentioned above, our circulation systems can respond to both short-term and long-term changes of tissue demand. The short-term response, which is the so-called metabolic flow regulation, is achieved by multiple means – regulating the heart blood pressure, changing vessel luminal diameters, and modulating the distribution of capillary flows [14]–[18]. Meanwhile, the long-term response, which is the chronic adaptation, also includes multiple structural changes in luminal diameters, vessel wall thicknesses, and micro-vessel densities [19]–[25]. Both short-term and long-term responses can benefit the efficiency of blood delivery and help to maintain the blood pressure in capillaries. Furthermore, the two responses appear to be similar in some aspects. For example, a short-term increase of blood flow can induce a dilation of blood vessels, whereas a long-term increase of blood flow can induce a structural increase of vessel luminal diameters [15], [16], [19]–[21].

The wall shear stress plays an important role in both metabolic flow regulation and chronic adaptation. On the one hand, the short-term change of luminal diameter is experimentally observed to be related to the wall shear stress: the vessel dilates when the blood flow increases and it contracts when the blood flow decreases, in such a way that the wall shear stress is maintained at a relatively steady level [14]–[17]. In these experiments [14]–[17], endothelial cells are found to be responsible for sensing the wall shear stress. On the other hand, a long-term increase or decrease of blood flow can also induce a structural increase or decrease of vessel liminal diameters [10]. It has been long discovered that there is a cubic relation between the vessel diameter and the blood flow within the vessel [26]. As is noted above, this relationship is later explained by Murray [27] by minimizing a cost function. Murray’s analysis suggests an optimal wall shear stress in the circulation system. Later, it has been further shown in experiments [7], [28] that the wall shear stress does not have large variations along the circulation.

Based on the above experimental observations [7], [14]–[17], [28], it has been assumed in previous studies that vessels can adapt their luminal diameters to maintain a preset wall shear stress [8], [30]. In general, this can be described as
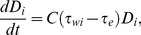
(4)where 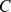 is a positive constant corresponding to the growth rate of diameters and 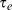 the expected uniform wall shear stress in the circulation. The term 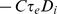 characterizes an intrinsic decreasing tendency due to cell death and other effects, and the term 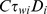 describes the increasing tendency stimulated by the wall shear stress. From experimental observations, it is suggested that the coefficient 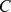 might be a constant for different sizes of vessel. However, it has not been sufficiently verified. In theoretic analysis, it does not have to be strictly a constant. A slightly varying coefficient 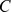 will not affect the stability of a vessel system. Therefore, we assume this coefficient 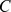 to be a constant in the following stability analysis.

The blood pressure also plays an important role in the metabolic flow regulation and chronic adaptation. It is directly related to the adaptation of vessel wall thickness by the circumferential wall stress. In general, the preset wall shear stress 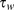 can be dependent on pressure, i.e., 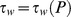. These effects have been shown to have no strong impact on the stability issue [8]. Therefore, we will not include these effects in our current work.

#### Stability of vessel system

We begin with a brief review of the stability analysis of some simple vessel systems for the above model. The first two cases have been considered in detail in the work of [30]–[32]. We recapitulate the main points of these results and emphasize some important issues related to the stability.

#### Case 1

**Figure 1:**
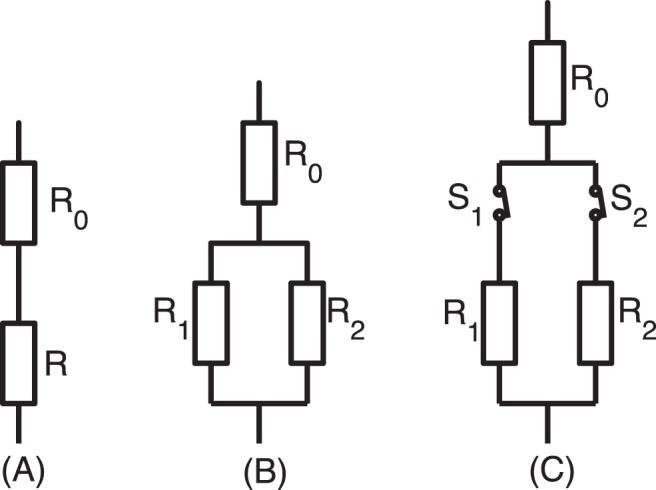
Illustration of simple systems in the vessel network. The icons are borrowed from the electrical circuit [30]. (A) A vessel is in series with a fixed resistance 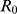. (B) The parallel part of two vessels is in series with a fix resistance. (C) The parallel part is controlled by the open-close switches.

In Fig. 1 (A), a vessel with adaptable diameter 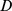 and fixed length 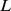 is in series with a fixed resistor 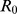. The pressure drop on the vessel system is fixed to be 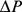. When the pressure drop on the vessel system is sufficiently large, the adaptation of the vessel with a fixed resistor in series has two equilibrium diameters, 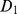 and 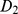 (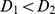). The larger equilibrium diameter, 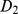, has been shown to be locally stable, namely, when the initial diameter is greater than 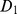, the diameter adapts to 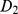 eventually. For the adaptation of a single vessel without fixed resistance in series, it is stable in the case of constant flow (fixed flow in the vessel), whereas unstable in the case of constant pressure (fixed pressure drop along the vessel).

#### Case 2

**Figure 2:**
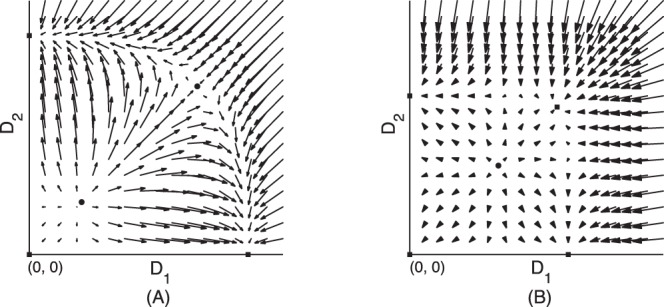
Illustration of adaptation in the phase plane. Squares denote the stable equilibrium states. Arrows starting at point 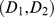 stand for the rate of change of the two diameters. (A) phase plane of a parallel vessel system. In the three stable equilibrium states, at least one diameter is zero, i.e., at most one vessel can survive. (B) phase plane of a parallel system with switches (fixed OTRs). There is a new stable equilibrium in which neither diameter vanishes, i.e., both vessels survive.

In Fig. 1 (B), two parallel vessels, with adaptable diameters and fixed lengths 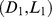 and 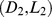, respectively, are in a serial connection with a fixed resistance 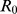. In this case, at least one of the diameters will decrease to zero eventually, due mainly to the fact that the vessel with a smaller value of 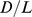 in a parallel system always has a smaller wall shear stress. Even if there are more vessels in parallel, at most one of the vessels can survive in the end. Similar to the instability of parallel vessels, this vessel adaptation process is not stable for a vessel network which contains parallel parts in their sub-components. The adaptation process is shown in phase plane in Fig. 2.

It has been realized in the works of [8]–[10], [33], [34] that the structural adaptation must meet the metabolic demand. From this point of view, a reference blood flow rate was introduced for each vessel and an additional metabolic stimulus was added to the adaptation system [8]–[10]. Metabolic stimulus is also introduced in the works of [33], [34] to study responses to the local state of the tissue. In these studies, the metabolic stimulus gives rise to a locally stable adaptation system.

In the following, we will discuss a possible physiological mechanism for the origin of the metabolic stimulus. In fact, the blood vessel system can meet the metabolic demand by metabolic flow regulation. As a result of the change in blood flow due to flow regulation, the wall shear stress in vessels is changed, resulting effectively in an extra stimulus in the adaptation. We will take into account the effects of metabolic flow regulation in our new adaptation model. In this model, we incorporate fluctuations of capillary flows in the form of an open-close switch of capillary flows.

## Results

### Analysis of Stability with an Open-close Process

In this section, we present the details of our model for adaptation of vessel systems and show that the short-term metabolic flow regulation of blood flow is able to stabilize the long-term adaptation of vessels after taking into account the effects of the open-close switch of capillary flows.

Fluctuations in the distribution of capillary flows are important in the metabolic flow regulation of blood flow [1]–[6]. As is reported in [5], capillaries are controlled in groups by single upstream terminal arterioles. Blood flow in a capillary group can be switched on or off by its upstream arteriole and possibly the post-capillary vasculature [4]–[6]. We model the phenomenon of fluctuations of capillary flow by an open-close switch and this switch process is sensitive to the state of the tissue, such as the concentration of O_2_, CO_2_, and K^+^. A low concentration of O_2_, or a high concentration of CO_2_ and K^+^ tends to relax smooth muscle cells of the terminal arterioles, allowing the blood to flow through the capillaries, whereas a high concentration of O_2_, or a low concentration of CO_2_ and K^+^ leads to contraction of smooth muscle cells, cutting off the blood flow in capillaries [1]–[4]. The variation in the number of flowing capillaries in response to change of tissue state is referred to as capillary recruitment. In other words, the open-close switch of capillaries can play a controlling role in regulating the blood flow in response to the tissue states. A graded contraction of arterioles leads to a decrease of blood flow in the downstream capillary group. In this case, the capillary flow is not closed completely but the effects in adaptation should be similar. We will first focus on the open-close switch of capillary groups in the following, and graded contractions will be discussed afterwards. Although the concept of capillary recruitment, which is widely accepted, is implemented in this work, we note that for regulation of blood flow in skeletal muscles during exercise, the role of capillary recruitment has yet to be clarified [35].

**Figure 3:**
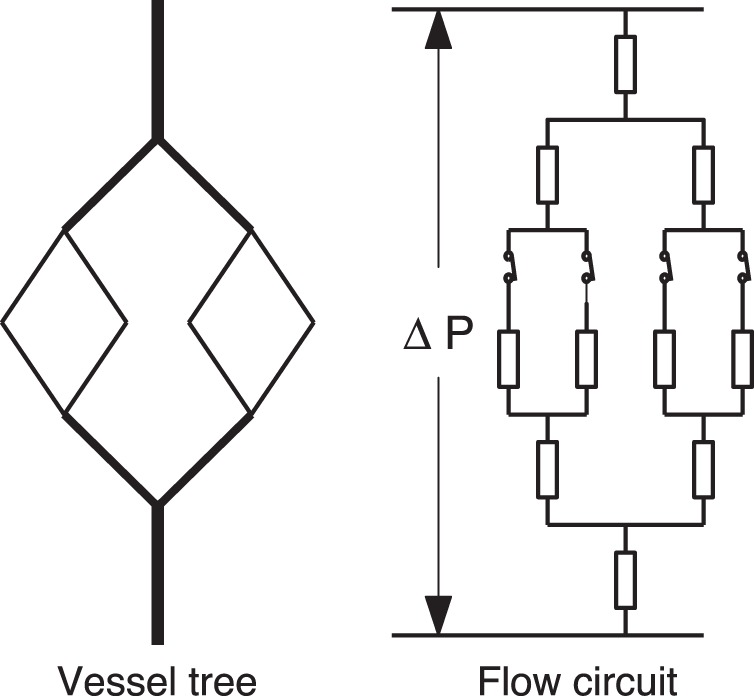
A vessel network and the corresponding electrical circuit. There is a switch at the inlet of each capillary group in the middle layer.

In order to incorporate into the adaptation model the effects of fluctuations in capillary flows, each capillary group in the vessel system is assumed to have two states – open and close. The resistance is calculated according to Eq. (3) when the capillary group is open, whereas the resistance is set to be infinite when the capillary group is closed. In comparison to the electrical circuit, the effect of the open-close switch is similar to that of an electrical switch. We make no modification on the adaptation equation (4) in our new model, but the blood flows in the vessel network are now controlled also by the open-close process (see Fig 3).

Clearly, the blood flow in an vessel network depends on the open-close state of the capillary flows. In our model, we assume the time-averaged stimulus of the wall shear stress is the effective stimulus for the increase of vessel diameter. The time scale of the open-close switch, which is on order of seconds, is much smaller than that of the vessel adaptation, which is on order of weeks [36]–[38]. This fact allows us to calculate the time-averaged stimulus by the average stimulus of all states of different open-close configurations. Therefore, we turn to a detailed description of the open-close switch. The Open Time Ratio (OTR), 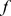, is introduced to characterize the mean effect of the open-close process on adaptation, where OTR is the percentage of the time in which the capillary flow in a group is switched on. The characteristic value of the OTR depends on tissue types and their states. When surrounding tissues are in their rest state, it has been estimated in experiments [37], [38] that the OTRs of capillary flows are about 0.2. This characteristic value can also be examined from a different perspective, namely, it can be regarded statistically as the characteristic percentage of capillary groups that are open, or the probability that a capillary group is in an open state. As will be seen below, it is conceptually convenient to regard the OTR as the open probability in the model. Although the open-close process of neighboring capillary groups may be weakly correlated due to mass diffusion in tissue, for simplicity, we will assume that the open-close switches are statistically independent. To develop intuition about the effect of the open-close switch and to obtain insight into the role of the switch in regulating vessel systems, we consider the stability of a parallel vessel system with switches. We assume that OTRs are uniform in this case. In our model, we will use a single vessel to represent a capillary group. The adaptation stability of parallel capillary groups may involve the stimulus that comes directly from the local tissue and will not be addressed in this work.

#### Case 3

In contrast to Case 2, as shown in Fig. 1 (C), each parallel part has a switch. Assume the OTRs of the two switches are 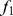 and 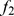, respectively. There are four different states of the vessel system under the independence assumption, as tabulated in Table 1, in which the state 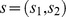 denotes the open-close states of the two capillary groups, where
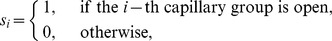
where 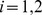, and 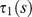 and 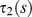 are the wall shear stress of the two capillary groups at state 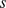, respectively. In the adaptation process, the effective stimulus of the fluctuating flow due to the switch among different states is assumed to be the average of the wall shear stress in our model. Therefore, the wall shear stress in the adaptation equation (4) is replaced by a weighted average of the wall shear stress over the four states according to their time ratio 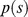


**Table 1:** Wall shear stress in different states.

state s	time ratio p(s)	*τ*_1_ (s)	*τ*_2_ (s)
(1, 1)	*f*_1_ *f*_2_	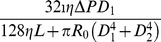	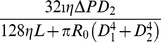
(1, 0)	*f*_1_ (1−*f*_2_)	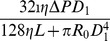	0
(0, 1)	*f*_2_ (1−*f*_1_)	0	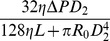
(0, 0)	(1−*f*_1_) (1−*f*_2_)	0	0

The state 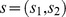 denotes the open-close states of the two capillary groups, where 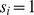 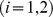 when the 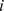-th capillary group is open and 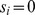 when it is closed. 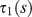 and 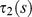 are the wall shear stresses of the two capillary groups at state 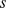, respectively.



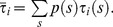
(5)In case of 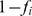 being much greater than 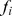, as shown in Table 1, the time ratio 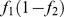 for the case in which only capillary group 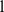 is open is much greater than the time ratio 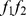 for the case in which both capillary groups are open. In other words, for a large portion of time, the two vessel groups are not parallel to each other, but directly in series with the fixed resistance 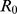. As a result, the stability behavior of the system should be similar to that of a serial system, which has been shown to be stable as summarized in Case 1. We note that the typical value of 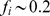. Therefore, our argument above can be valid approximately.

The adaptation process for the case 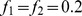 is illustrated in the phase plane as shown in Fig. 2 (B). Arrows starting at the point 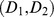 stand for the rate of change 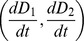, which is obtained using Eq. (4) in the average sense, i.e., the wall shear stress is obtained using Eq. (5). Fig. 2 (B) also suggests the stability of the equilibrium state for co-existing parallel vessels with switches. A detailed analysis of the stability for a symmetric parallel system is presented in Section Methods – when the OTR is smaller than a critical value, 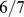, and the pressure drop on the vessel system is sufficiently large, the adaptation has a stable co-existing equilibrium state.

Now we turn to consideration of the effect of the open-close switch on the stability of vessel trees. The vessel tree is assumed to be in series with a fixed resistance. We further assume that there is a constant pressure drop along the vessel system. In our model, the “leaves” (i.e., the smallest vessels) of the vessel tree represent capillary groups that have two states – open or close.

For a vessel system with 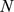 capillary groups, there are 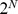 different states of configurations. In this case, the wall shear stress in the adaptation equation (4) is replaced by its weighted average over all the different states using Eq. (5). In this case, the state variable 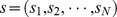 denotes the state of all the capillary groups
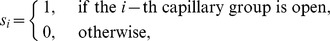
and 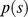 is the probability of the state 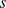




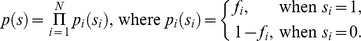



Clearly, the average blood flow rate in the vessel tree is
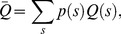
(6)which allows us to compute the average wall shear stress as



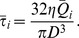
(7)Using similar arguments to those in Case 3, one can see that when a vessel tree is small (i.e., the number 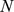 of capillary groups is small), the adaptation of vessels allows the parallel “crowns” (i.e., subtrees of the vessel tree, as shown in Fig. 3) to co-exist. For example, when all the OTRs are set to be 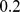, a 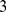-level complete binary vessel tree is locally stable in our simulation of vessel trees according to the adaptation equation (4) (for details, see below). Essentially, the stability of parallel crowns also arises from the serial property induced by the open-close switches. However, when the number of capillary groups becomes large (e.g., there are 16 capillary groups in a 5-level complete binary vessel tree), the results of our simulations indicate that the adaptation of the system becomes unstable. In fact, the larger crowns are less likely to be in serial connections with the constant resistance, and they become effectively parallel to each other, because there are open capillary groups in both crowns for majority of time. As a result, the adaptation of a large vessel system with fixed OTRs is unstable. This conclusion is similar to that of Case 2. In our simulations for large vessel systems, we find that the vessel diameters are adapted to a state nearly satisfying Murray’s law at first, then the vessels in one of the largest sub-trees that has a smaller wall shear stress are narrowed almost proportionally. Eventually, all the vessel diameters of this crown becomes zero (we will refer to this phenomenon as *global degeneration*). Furthermore, one of the daughter crowns of the surviving sub-tree will become also degenerate if the size of the crown is still large. Finally, only a small vessel crown (e.g., a four-level vessel crown) can survive during the adaptation process of the vessel system.

So far, we have only taken into account the open-close switch by a fixed value of the OTRs in our discussion above. Therefore, the effects of metabolic flow regulation are not yet incorporated into our model, which allows the open-close switch to modulate blood flows according to tissue demand. Next, we model the effects of metabolic flow regulation through a constitutive relation of the OTR with the blood flow, and discuss the effect of this metabolic flow regulation in the adaptation of large vessel systems. As will be shown below, the adaptation of large vessel systems becomes stable after incorporating this type of metabolic flow regulation, that is, through the coupling between the blood flow and the OTRs.

As is mentioned above [1]–[6], [18], when the average flow in a capillary group decreases, the concentration of O_2_ (CO_2_) in the tissue over which the capillary group perfuses becomes lower (higher). As a result, the capillary groups automatically opens more, i.e., the OTR increases. In this sense, the micro-vessels can modulate the blood flow in response to the local tissue demand. Therefore, we introduce a reference blood flow rate for each capillary group (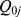, where 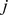 is the index of the capillary group). We define the normalized flow rate 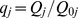, where 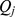 is the blood flow in the 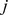-th capillary group. We model the OTR, 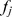, as a function of 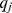. We define the sensitivity, 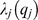, of the 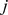-th switch as
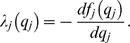



According to the metabolic flow regulation behavior discussed above, an increase of blood flow results in a decrease of OTR. Therefore, we have
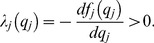
(8)


As we mentioned above, the stability of the adaptation of two large parallel vessel crowns are similar to that of two parallel vessels as summarized in Case 2. Hence, we re-examine this issue using our model of metabolic flow regulation, i.e., by coupling the change of the OTRs with the change of blood flow rate. We will describe important relevant results of the parallel vessel system as shown in FIG. 1 (B). These results will facilitate our understanding of the stability property of large vessel trees.

#### Case 4

We consider the vessel system shown in Fig. 1 (B), in which the parallel effective vessels represent parallel vessel crowns. For simplicity, an initial perturbation 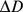 is introduced to only one effective vessel (vessel 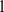 in Fig. 1 (B)) of the parallel part, where 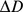 is the deviation from the equilibrium diameter 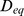. Since the resistance of the blood vessel is changed by the perturbation, the blood flow in it also changes, giving rise to a change in the OTRs of the capillaries in the vessel crown. As a result, the change in the resistance of the effective vessel has a component 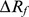 that is induced by metabolic flow regulation. Note that, for a positive (or negative) 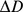, the change of blood flow is positive (or negative). Thus the change of resistance 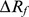 induced by metabolic flow regulation has an opposite sign to 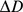. From Eq. (3), the resistance 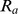 of the vessel after the perturbation can be written as
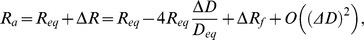
where 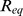 is the resistance of the vessel at the equilibrium state. From our discussion above, we can see that the change of resistance 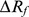 due to metabolic flow regulation has an opposite sign to the change of resistance 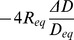 due to the perturbation. In other words, the metabolic flow regulation-induced change of resistance 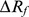 gives rise to a compensation to the change of resistance arising from the perturbation. Hence, a compensation to the flow in the vessel. Clearly, in the critical case that 
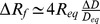
, the compensation is so large as to maintain the blood flow at the value of equilibrium. Hence, the system is stable, which is similar to the case of constant flow rate as we mentioned in Case 1. This suggests that the system should be stable as long as the compensation is sufficiently large, even if it does not fully compensate the blood flow.

In the following, we describe the condition for 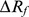 that renders the system stable. For Fig. 1 (B), according to the Kirchoff’s law, we can obtain the blood flow and wall shear stress for vessel 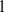


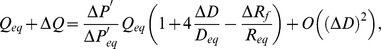


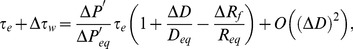
(9)where 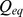 is the blood flow in vessel 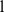 before perturbation, and 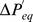 and 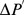 are the pressure drop on the parallel part before and after the perturbation, respectively. Due to the perturbation, the blood flow in vessel 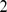 also changes because the blood pressure changes from 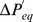 to 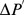, which can also induce metabolic flow regulation. However, the change of blood pressure in large crowns due to the perturbation is generally very small. Therefore, we ignore the change of resistance in vessel 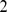, and obtain the wall shear stress



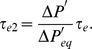



As a consequence of the argument employed in Case 2, we observe that the key point for the system to be stable is that the smaller one of the two parallel vessels should have a greater wall shear stress. Therefore, on the order of 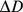, we obtain approximately the necessary condition for the system to be stable
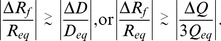
(10)


We can see that according to Eq. (10), in order to stabilize the adaptation of parallel systems, at least 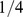 of the change in resistance (or in blood flow) should be compensated. In the work of [8], a flow-dependent metabolic stimulus is introduced to stabilize the adaptation. A similar analytical result on the metabolic stimulus obtained in the work of [8] provides a lower bound of the metabolic stimulus for adaptation stability.

We will further examine the condition (10) by direct numerical simulations after we discuss the relation between 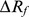 and the sensitivity 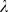 (see below). The result of our simulations suggest that the conditions (10) are approximately valid.

When the capillary flow is regulated gradually, the strength of the regulation and the probability for the graded regulation to switch on may respond to the local tissue states. This graded regulation also induces a change of the effective resistance. Therefore, the compensation effect of effective resistance of graded regulation is similar to that of open-close switch. When the compensation is large enough, the adaptation process of large vessel systems also becomes stable with graded regulation of capillary flow.

Aside from the metabolic flow regulation as we modeled here, in general, other means of metabolic flow regulation, such as responses to the wall shear stress and to blood pressure [15], [16], have similar effects in the compensation of blood flow, thus helping to maintain a stable adaptation. For example, when a vessel is initially smaller than its equilibrium state, the resistance of the vessel is larger than that of the equilibrium state. Therefore, the blood pressure in its downstream vessels becomes lower. The metabolic flow regulation to the decreasing blood pressure results in a non-structural dilation of the vessels, leading to an increase of blood flow as a compensation. This similar compensatory effect can likely help to stabilize the adaptation of the system.

Finally, for later discussions, we mention other factors that can affect the OTRs. These factors include the tissue activity, 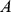, the capillary density, 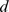, (the number of capillaries in unit volume of tissue) and the partial pressure, 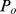, of Oxygen in the blood. When the tissue is in an excitory state (e.g., when the muscle tissue is at work), the tissue activity 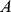 is larger than the rest state, and the consumption of Oxygen in tissue is faster. According to the response of the open-close process to the tissue state as discussed at the beginning of this section, the OTR becomes greater. Therefore, we have
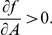



Similarly, we further have
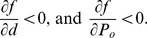



For an animal living at high altitude plateau, the partial pressure of Oxygen in the air is low, leading to a low partial pressure of Oxygen in its blood. As a result, the OTRs becomes greater, which helps to deliver more blood to the tissue. From this point of view, the multi-function of the open-close process is not only important in maintaining the stability of the vessel adaptation, but also important in the acclimatization to living conditions.

Now we turn to comparison of the new model with previous models. In the work of [8]–[10], a reference blood flow rate for all vessels (including the high order vessels) is introduced. A metabolic stimulus related to the flow rate is incorporated into the adaptation process. This additional stimulus can stabilize parallel vessel systems. In the modeling work of vascular remodeling in tumors [33], [34], the large vessels that are distant from the tumor are not allowed to collapse, thus the stability issue of these vessels are avoided. Vessels inside the tumor are adapted according to the local tissue Oxygen concentration, which acts as a metabolic stimulus and stabilizes the adaptation process of small vessels. In both models, metabolic stimulus is crucial for the stability in adaptation process. In contrast to the works of [8]–[10], in our new model, the reference blood flow rate is introduced only for capillary groups. We do not explicitly introduce metabolic stimulus. The short term metabolic flow regulation according to the capillary flow is incorporated to modulate the blood flow. As a result of the change in blood flow, wall shear stresses in large vessels also change. The change of shear stress results in an additional effective stimulus, which stabilizes the adaptation of large vessels. For small vessels near the tissue that they superfuse, additional metabolic stimulus may come directly from the tissue and can be important in their adaptation. In particular, such stimulus may be important in the stability of parallel capillaries in a group.

In the modeling work of [39], capillaries are generated or removed according to the tissue states at each time step, and the diameters of higher order vessels are modified according to Murray’s law. Therefore, an infinite adaptation speed of the vessel diameters and fixed flow rates in capillaries in each step are assumed in this model. As summarized in Case 1, the constant flow case is stable. In other words, the change of the blood flow is assumed to be fully compensated by the metabolic flow regulation process. As discussed above, when the compensation of the change of blood flow is partially compensated (more than 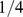 of the original change) by the metabolic flow regulation, it is sufficient to render a stable adaptation.

### Numerical Results for Stability of Vessel Trees

In Section Methods, we provide a simple but efficient numerical method for the simulation of large vessel systems. The simulation is helpful for understanding the stability behavior of the adaptation of large vessel trees.

#### Vessel System

We simulate the vessel adaptation of an 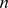-level M-tree. As defined in Section Methods, an M-tree is a complete symmetric binary vessel tree, whose diameters satisfy Murray’s law and the vessel length of each vessel is proportional to its diameter. We add perturbations at random to the initial value of diameters. The constant 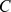 and the preset wall shear stress 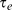 in Eq. (4) are assumed to be uniform in the vessel tree, and the pressure drop 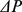 along the vessel system is fixed at a constant value. The sensitivity of the OTRs is assumed to be a constant, which is the same for all the switches. Hence, the constitutive relation of the OTRs is
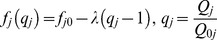
(11)where 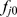 is the reference OTR, 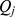 and 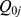 are the flow rate of the 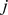-th capillary group and its reference value, respectively, and 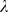 is the constant sensitivity. When 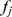 is greater than 1 (or less than 0), the OTR is simply set to be 1 (or 0).

**Stability**. Now we consider the validation of the condition (10) for stability of parallel vessel crowns. Note that, for large vessel trees, we don’t have the value of 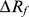 and 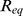 yet, since there are too many different states.

In order to consider the average effect of the many different states, we define the effective resistance, 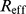, of a vessel tree (or a vessel crown) by
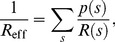
(12)which is a weighted harmonic mean resistance of all different states. If the pressure drop 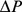 on the vessel tree (or a vessel crown) were independent of the state 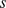, this effective resistance could be used to compute the average blood flow in the root vessel by



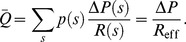
(13)The pressure drop generally depends on the state. For a large vessel tree, however, it varies within a relatively small range for most states (see detailed discussion in Section Methods). This implies that Eq. (13) holds approximately and allows us to compute the effective resistance of a large vessel tree (crown) approximately by that of its two daughter crowns
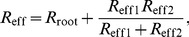
(14)where 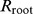 is the resistance of the root vessel of the vessel tree (crown), and 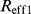 and 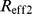 are the effective resistance of its daughter crowns respectively. The validity of Eq. (13) suggests that we can use the effective resistance 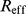 to replace 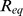 and calculate 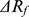 in the stability condition (10).

**Figure 4:**
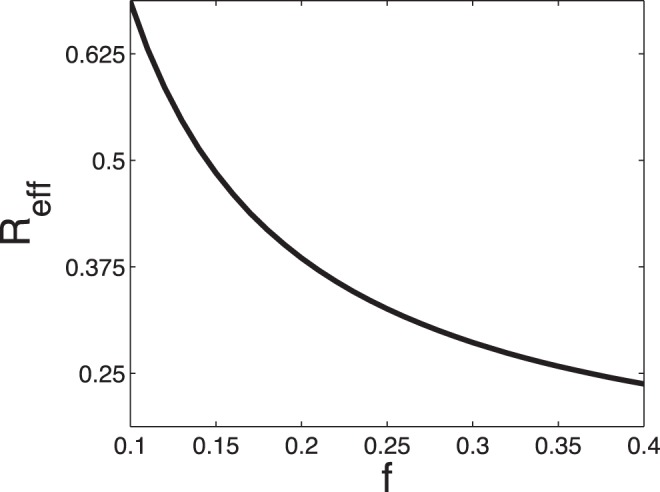
Effective resistance of a 5-level M-tree. The resistance and its derivative are decreasing functions of the OTR. The values in the ordinate are normalized by the resistance of a capillary group.

Next we consider the relation between 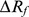 and the sensitivity 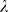. As discussed in Section Methods, when the vessel crowns are large, we can compute the effective resistance recursively according to Eq. (14), until the daughter crowns are sufficiently small (e.g., a 5-level tree). In other words, we can compute the effective resistances of small vessel crowns by definition, then use these effective resistances to compute the resistances of large vessel crowns and eventually the entire vessel tree. For a 5-level M-tree with uniform OTR 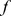, the profile of the effective resistance, 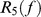, of the 5-level M-tree is shown in Fig. 4.

Using Eq. (14), we can recursively compute an approximate effective resistance, 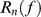, of an 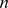-level (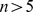) vessel crown
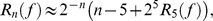
where 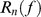 is normalized by the resistance of the capillaries. Therefore, from Eq. (11), we have



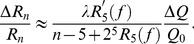
(15)The condition (15) leads to the following condition that the sensitivity 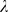 satisfies.
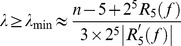
(16)for a locally stable 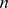-level M-tree.

In our numerical simulations, we first add 5% random perturbations on the vessel diameters of a 7-level M-tree initially
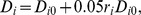
where 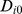 is the vessel diameter before perturbation and 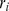 is a random number uniformly distributed in the interval 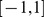. Then we evolve the vessel diameters according to the adaptation equation (4). To maintain the stability, the lower bound of the sensitivity 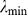 estimated from Eq. (16) is about 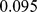 for 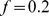 (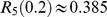 and 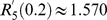). In our simulation, the adaptation is stabilized when 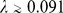. This is in good agreement with the estimate value. Therefore, the numerical simulation confirms that the condition (10) is valid as an approximate stability condition for large vessel trees (vessel crowns).

It can be seen from Eq. (16) that the lower bound of the sensitivity is an increasing function of the level 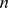 of the vessel tree. Therefore, a sensitivity 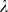 that can stabilize a relatively small vessel tree may not be able to stabilize a large vessel tree. For example, with 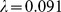, a 7-level vessel tree is stable, but an 8-level vessel tree is not stable. When the sensitivity 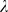 is not large enough, the stability behavior of the adaptation is similar to the case in which the OTRs are fixed, i.e., there is also a global degeneration. For greater sensitivity 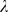, the surviving vessel crown can be larger.

The total number of capillaries in our body is of an order of 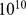, which corresponds to a lower bound of the sensitivity 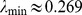 estimated from the M-tree. The modeling study in the work of [40] shows that 20–30% increase in Oxygen demand results in an increase by 25–42% of perfused capillaries. This result is in good agreement with the experimental data in the works of [41], [42], which shows that a comparable increment in Oxygen demand is associated with an increment of capillary filtration coefficient at about 18–43%. According to these studies, we can estimate the sensitivity of the open-close switches, which is about 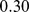. It is worthwhile to point out the following: First, for animals that have bigger size than human beings, the lower bound of sensitivity for adaptation stability is also greater; Second, other means of metabolic flow regulation can have similar effects on the compensation of blood flow. This can help to decrease 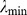; Third, further experiments which aim directly at the measurement of the sensitivity hopefully can help us to obtain a more accurate value of the sensitivity.

#### Micro-Vessel rarefaction

Micro-vessel rarefaction [43]–[48] is a phenomenon in the adaptation of real vessel networks, i.e., some *small* arterioles, *small* venules, and capillaries become degenerate in many different vessel crowns. Micro-vessel rarefaction is closely connected with the pathogenesis of hypertension [43]–[45]. Micro-vessel rarefaction induces an initial increase of heart blood pressure in a hypertension patient, then the adaptation processes generate a vicious circle between the increased blood pressure and increased peripheral resistance [43]–[45], resulting in thickened vessel walls and narrowed vessel lumens. In order to understand the pathogenesis of hypertension, it is important [43], [49] to understand the origin of rarefaction in hypertension patients.

Furthermore, experimental observations [46]–[48] suggest that decreased tissue activity can also result in micro-vessel degeneration, whereas increased tissue activity can prevent micro-vessel degeneration or even increase the number of micro-vessels. It is also suggested [25], [50], [51] that a low partial pressure of oxygen in arterial blood can slow micro-vessel rarefaction or prevent rarefaction from occurring in the vessel system. Based on our discussion on the behavior of the open-close process, decreased tissue activity results in lower OTRs, whereas increased tissue activity, or low partial pressure of oxygen, results in greater OTRs. This leads us to hypothesize a scenario that, a global decrease of OTRs (which means all OTRs in the vessel network decrease) can induce micro-vessel rarefaction, whereas a global increase of OTRs can help to prevent micro-vessel rarefaction.

**Figure 5:**
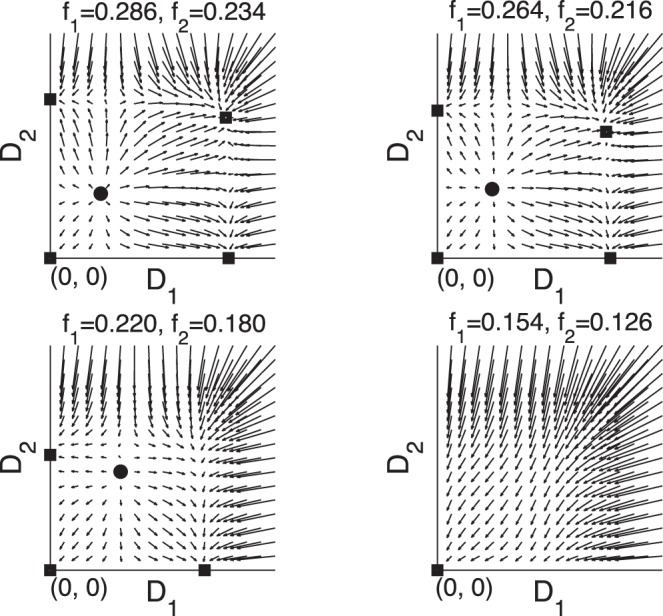
Global decrease of OTRs leads to vessel rarefaction. For the parallel system shown in Figure 1 (C), the OTRs of the two switches are different. When the OTRs decrease with the ratio 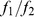 fixed, the stable co-existing equilibrium state becomes unstable. Similarly, for complex vessel systems, when there is a global decrease of the OTRs, the vessels with small OTRs may become degenerate and micro-vessel rarefaction occurs. If the OTRs decrease too much, there can be a global degeneration of the vessel system.

This scenario is also confirmed in our numerical simulation. In our simulation, we made a 10% random perturbation to the reference blood flow rate of capillary groups 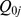 (or to the reference OTR 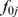). As a result, some of the capillaries have smaller OTRs than the others. In the simulation, in different vessel crowns, we have observed degeneration of capillary groups that have small OTRs, but the entire vessel crowns are stabilized by a sufficiently large sensitivity 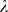 (e.g., 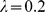 for the 7-level M-tree). Furthermore, as we decrease the reference blood flow rate proportionally throughout the entire system, more vessels become degenerate, i.e., a global decrease of the OTRs can induce rarefaction. In FIG. 5, we show the stability behavior of a parallel system. The two capillary groups of the system have different OTRs. As we decrease the OTRs of the two capillary groups proportionally, the co-existing stable equilibrium may disappear. This example suggests that a global decrease of the OTRs is a possible origin of the micro-vessel rarefaction. This is consistent with the experimental observations [25], [46]–[48], [50], [51]. Because there is also micro-vessel rarefaction in patients who are susceptible to hypertension, it may be important to study the behavior of OTRs in those patients.

## Discussion

We have proposed a model of vessel adaptation taking into account the minimal effects – the wall shear stress, the preset decreasing tendency, and the open-close process of capillary groups. The central assumption of our model is that the open-close switch of capillary flow responds to local tissue states, and enables the vessel system to meet the local tissue demand. An OTR is defined for each capillary group to characterize the mean effect of the open-close switch. For a large vessel system, the adaptation stability is achieved when the sensitivity of the open-close switch to blood flow is sufficiently large. Other means of metabolic flow regulation can also help to achieve the adaptation stability. Comparing to the previous works of [8]–[10], [33], [34], our model does not introduce additional reference flow rate and metabolic stimulus in large vessels. In our model, such an additional stimulus is induced by the change of blood flow (hence, wall shear stress) through the metabolic flow regulation of the low level vessels. In other words, it is the short term response (metabolic flow regulation) of the vessel system that stabilizes its long term adaptation. We point out that our model allows for the degeneration of vessels through vessel adaptation.

Our numerical studies on large vessel trees suggest that a global decrease of OTRs can lead to micro-vessel rarefaction. This result is consistent with the existing experimental observations. Because the pathology of hypertension also involves the micro-vessel rarefaction process, it might be important to study the behavior of OTRs in patients who are susceptible to hypertension.

The effects manifested in our minimal model are already able to achieve the adaptation stability of arterial trees by responding to tissue demand. However, other effects can also be important in the vessel adaptation. For example, (1) although we have idealized the flow regulation by the on-off control of capillary flows, the effects of flow regulation by arterioles upstream of the terminal arterioles, which modulate flow without shutting it off, can also be important for maintaining the adaptation stability. (2) The circumferential wall stress is important in remodeling the wall-to-lumen ratio [10], [11]. Chronic high pressure may result in hypertrophy, which is also a characteristics of hypertension [10], [11]; (3) The existence of parallel capillaries and capillary loops in a capillary group suggests the existence of additional possible stimulus in the capillary bed. This additional stimulus may directly respond to local tissue states; (4) The generation of new vessels [52] is important for the growth of individuals and is important for chronic increase of tissue activity.

## Methods

### Stability of the System in Case 3

For simplicity, we assume that in Fig. 1(C) two capillaries have the same vessel length 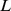 and the same fixed OTR 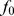. The mean wall shear stresses of the two vessels are obtained from Table 1 by a weighted sum
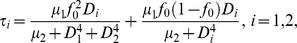
where 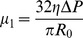 and 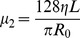. At the co-existing equilibrium state, the two vessels have the same diameter 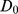, which satisfies



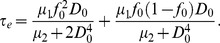
(17)The Jacobian matrix at the equilibrium can be obtained from the adaptation equation (4)
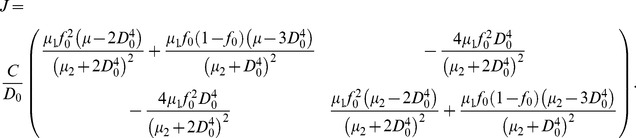
(18)


The local stability of the co-existing equilibrium state implies that both eigenvalues of the Jacobian matrix (18) are negative. This is equivalent to
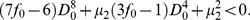
(19)


For 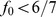, if 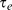 is sufficiently small, the solution of 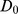 obtained from Eq. (17) satisfies the condition (19), and the co-existing equilibrium state for the two vessels is stable.

### Numerical Methods

Here, we provide a simple but efficient numerical method for the simulation of the adaptation of vessel trees. The numerical method can be further generalized to study more complicated models and more complex vessel networks.

The general numerical methods for evolving ordinary differential equations, such as the Euler method and the Runge-Kutta method, can be directly applied to the adaptation system described by Eq. (4). At each time step, for a general vessel network, with all the vessel diameters (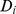) known, we can obtain the wall shear stress (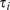) needed in Eq. (4) for all the vessels as follows: First, we compute the resistance, 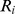, of all vessels by using Eq. (3), using the known diameters, 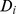; Second, using Kirchoff’s law, we obtain a system of linear equations for the blood flows 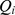 in the vessels and the blood pressures 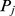 at the vessel junctions. The coefficients of the linear equations contain only resistances of the blood vessels. We solve the system of linear equations for the blood flows, 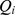, and blood pressures, 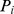, of all vessels; Third, now with blood flows 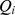 and diameters 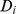 known, we can compute the wall shear stress, 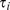, by using Eq. (2). Then, we can evolve one time step to obtain the diameters at the next time step using the adaptation equation (4).

**Figure 6:**
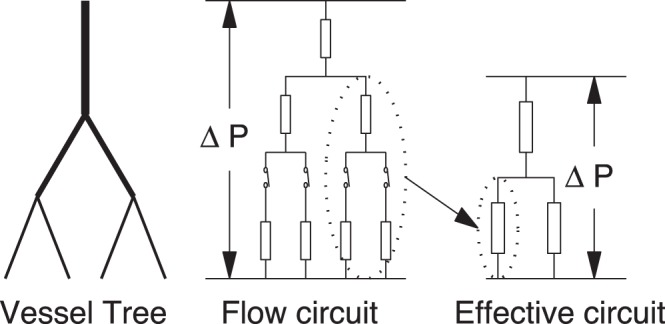
A vessel tree, its flow circuit and its effective circuit. In the simulation, in order to compute blood flows in all the vessels, small vessel crowns are replaced by their corresponding effective resistances.

When the vessel network has a tree structure as shown in Fig. 6, there is a simple way to compute the wall shear stress: by using Kirchoff’s law, we first recursively compute the resistance of all vessel crowns by their daughter crowns
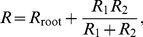
(20)where 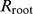 is the resistance of the root vessel of the crown. 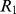 and 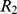 are the resistances of its two daughter vessel crowns, respectively. Using the resistance of the entire vessel tree, which is obtained through the above recursive process, we can obtain the blood flow in the root vessel of the tree. As can be seen directly from Kirchoff’s law, the blood flows in the daughter crowns of the root vessel are



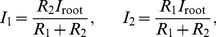
(21)respectively. Then, Eq. (21) allows us to compute the blood flows in all vessels recursively.

When we take into account the effect of the open-close switch, each capillary group in the vessel tree has two states – open and close. In this case, the wall shear stress in the adaptation equation (4) is replaced by the weighted average value in Eq. (5).

When the vessel tree is small, i.e., the level number 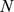 is small, we can directly compute the average wall shear stress using all the terms in Eq. (5). However, when 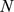 is large, the number of states is too large to numerically evaluate Eq. (5) efficiently, since there are 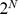 different states. Therefore, we need to devise further a numerical algorithm for computing 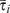 efficiently.

Clearly, we can compute the average blood flow rate in the vessel tree (or vessel crown) as
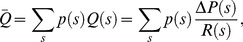
(22)where 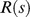 is the resistance of the vessel tree at state 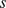. If pressure drop 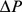 on a vessel crown were independent on the state 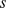, the effective resistance defined in Eq. (12) can be used to compute the blood flow by using Eq. (13). For small vessel crowns, we can compute the effective resistance exactly by using Eq. (12). However, for large vessel crowns, the exact evaluation of Eq. (12) has the same numerical difficulty as Eq. (22). To overcome this difficulty, we use the following approximate method instead of Eq. (12) to evaluate the effective resistance



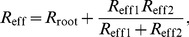
(23)where 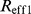 and 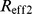 are the effective resistance of the daughter crowns or a root vessel, respectively. Eq. (23) is constructed by an analogy to Eq. (20), aiming at a recursive evaluation of the effective resistance for large crowns. In general, Eq. (23) is not exact, mainly because the pressure drop across the parallel part depends on the state. If the pressure drops on the daughter crowns were the same for different states, Eq. (23) would be exact. As can be expected, if the pressure drops do not have a strong dependence on the states, one can use Eq. (23) to compute the effective resistance 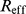 approximately. Fortunately, when two parallel crowns are large, the probability of extreme states in which nearly all the capillary groups are open or nearly all are close is very small. As a result, for most states, the blood pressure across the parallel part varies within a relatively small range. By this argument, Eq. (23) holds approximately for large vessel crowns.

The error between the exact effective resistance obtained via Eq. (12) and the approximate resistance obtained by Eq. (23) by using the exact effective resistance of its two daughter crowns, is shown below in detail. This error becomes very small when a vessel crown is large. For example, for a vessel crown with 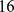 capillaries (both of its two daughter crowns have 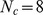 capillaries), the relative error is about 1% for a typical vessel tree with typical values of OTR.

Using Eq. (23), we can invoke the following numerical method to compute the wall shear stress for a large vessel tree (see Fig. 6). First, we compute the exact effective resistance of small crowns, each of which has 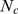 capillaries; Second, we replace the vessel crowns by effective vessels with the same effective resistance. This allows us to compute blood flow and wall shear stress in the large vessels and the effective vessel approximately, by the recursive process discussed at the beginning of this section; Third, we use the blood flow in the effective vessel to compute the wall shear stress in small crowns. Using this numerical method, for a large vessel tree with 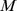 crowns, each of which has 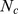 capillaries, the computational cost of each time step is reduced from 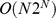 to 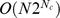, where 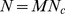 is the total number of capillaries.

### Error Analysis in Evaluating the Effective Resistance

Here we consider the computational error when we use Eq. (23) to calculate the effective resistance for both symmetric (M-tree) and asymmetric (Fibonacci Vessel Tree) binary vessel trees.

#### M-Tree

Let 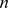 be the level number of the vessel tree. An 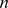-level M-tree is a complete symmetric binary vessel tree, whose diameters satisfy Murray’s law and the length of each vessel is proportional to its diameter. The capillary (group) number and the vessel number of the 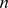-level M-trees are shown in Table 2.

**Table 2:** Vessel numbers and capillary numbers of M-trees.

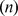 F-tree level	1	2	3	4	5
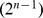 capillary number	1	2	4	8	16
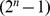 vessel number	1	3	7	15	31

For this tree, the diameters and resistances satisfy
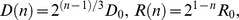
where 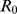 is the resistance of the capillaries. Using Eq. (23), the effective resistance of the 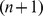-level vessel tree 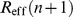 is obtained from that of its two 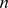-level crowns



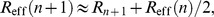
where 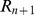 is the resistance of the root vessel of the 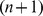-level tree. At the same time, we can directly calculate the effective resistance from the definition. In order to compare the values obtained by these two methods, we define the relative error as



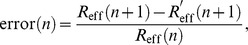
 where 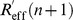 is the exact effective resistance obtained from the definition. The numerical results of the relative error in percentage are shown in Table 3.

**Table 3:** Relative errors in percentage for the effective resistance of M-trees.

OTR	error(1)	error(2)	error(3)	error(4)
0.15	20.42	6.15	2.33	0.94
0.2	18.95	5.51	2.00	0.76
0.3	16.08	4.34	1.45	0.52

#### Fibonacci vessel tree

Since the real vessel tree is not symmetric, to consider the asymmetric case we define a Fibonacci tree (F-tree): a 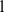-level F-tree is a capillary with diameter 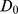; a 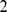-level F-tree is a symmetric vessel tree with two daughter 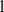-level F-trees; and an 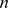-level F-tree (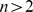) has an 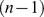-level left daughter F-tree and an 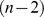-level right daughter F-tree. The tree is also assumed to satisfy Murray’s law. We have Table 4 for the F-trees.

**Table 4:** Vessel numbers and capillary numbers of F-trees.

F-tree level	1	2	3	4	5	6	7
capillary number	1	2	3	5	8	13	21
vessel number	1	3	5	9	15	25	41

Similarly, the relative error is defined as
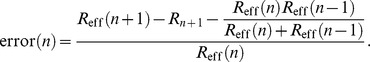



We tabulate the relative errors (in percentage) in Table 5.

**Table 5:** Relative errors in percentage for the effective resistance of F-trees.

OTR	error(2)	error(3)	error(4)	error(5)	error(6)
0.15	8.89	3.57	1.65	0.75	0.36
0.2	8.11	3.16	1.44	0.65	0.30
0.3	6.65	2.48	1.08	0.47	0.22

The above error is that of resistance, which also represents the error of the blood flow in the root vessel of the two crowns. Since the position of the capillaries in the tree is also asymmetric, there is an additional error due to the asymmetry, as in Table 6 (maximum error in percentage of all capillaries).

**Table 6:** Additional error due to asymmetry in percentage for the effective resistance of F-trees.

OTR	a error(2)	a error(3)	a error(4)	a error(5)	a error(6)
0.2	0	1.65	0.61	0.48	0.21

In conclusion, the relative error in computing the effective resistance using (23) decreases as the capillary number of the crown increases. When a vessel has 8 downstream capillaries, the relative error is about 1 for corresponding M-tree and F-tree.
